# Science-Economy-Technology Concordance Matrix for Development and Implementation of Regional Smart Specializations in the Silesian Voivodeship, Poland

**DOI:** 10.1155/2015/126760

**Published:** 2015-12-01

**Authors:** Adam Smoliński, Jan Bondaruk, Magdalena Pichlak, Leszek Trząski, Elżbieta Uszok

**Affiliations:** ^1^Department of Energy Saving and Air Protection, Central Mining Institute, Plac Gwarkow 1, 40-166 Katowice, Poland; ^2^Department of Water Protection, Central Mining Institute, Plac Gwarkow 1, 40-166 Katowice, Poland; ^3^Faculty of Organization and Management, Silesian University of Technology, Roosevelta 26 Street, 41-800 Zabrze, Poland

## Abstract

The regional smart specializations include the innovative activities within a common science-economy-technology sector, which open the opportunities to gain a competitive advantage. The original procedure of science-economy-technology concordance matrix development on an example of smart specializations of the Silesian Voivodeship was presented in the paper. The procedure developed includes recognition of the research and economic components of the regional smart specialization and the connection between the economic components of the regional specialization and the technological innovation through the international patent classification. It also comprises recognition of key enabling technologies (KETs) and high technologies (of high R&D intensity) other than KET in the economic and technological dimensions of innovation as well as the high R&D intensity services in the economic dimension of innovation. The in-depth expert analyses with the application of the Delphi method were also taken into account. The methodological approach developed and the visualization method applied are both of cognitive and practical importance since they contribute significantly to the creation of efficient development policies, to the enhancement and facilitation of cross-sectoral cooperation, and to the focusing on the fields of key importance in terms of the competitive advantage of a region.

## 1. Introduction

The continuous creation and implementation of innovative solutions, both technical and organizational, through planning and realization of long-term activities in the field of the strategic regional economic branch have been identified as the key elements for a region to succeed in the technological development and economic growth [1–3]. The Directorate-General for Regional and Urban Policy presented in 2011 the guidelines for the European, regional, third-generation innovation strategies concerning the development and boosting of the regional smart specializations [4, 5]. The EU expectations regarding European regions striving for the establishment of the regional smart specializations have been detailed in the document. This concerns principally the identification of several key priority investments of entrepreneurial potential in one or more regional smart specialization areas and requires close cooperation between science and business sectors focused on the creation of regional and international clusters [6–8]. This, in turn, involves creating a space for diverse, cross-sectoral connections in the supraregional networks formed within the clusters.

The Silesian Voivodeship is located in Southern Poland, in the region of Upper Silesia. It is a highly industrialized region, but the dominant heavy industry does not facilitate a strong and competitive regional economy or the growth of small and medium enterprises. Nevertheless, the potential of the region in terms of traditional industries and new services is undeniable. The companies providing niche products emerged in the posttransformation period, joining smoothly the global supply chain along with the enterprises operating in traditional regional sectors (coal mining, metallurgy, and power engineering). The changing economy sector imposed the adjustment of the R&D sector offer to the new market demands and increased its international activity within transnational research and expert networks.

The initial document, at the regional level, indicating the strategic goals of the innovation process enhancement was the “Regional Innovation Strategy of the Silesian Voivodeship for Years 2003–2013” [9], recently updated for years 2013–2020 [10]. It aimed at the creation of the effective support tools for the innovation, understood as the transfer of knowledge to small and medium enterprises of all sectors of the economy. The identified technologies of key importance in terms of the regional innovation development included energy and environmental technologies, information and communication technologies, and manufacturing and processing technologies as well as biotechnology, including bioengineering and biomedicine. The detailed analysis of the technological fields, strategic in terms of the regional innovation development, was performed in 2006 within the technological foresight study entitled “Priority Technologies for the Sustainable Development of the Silesian Voivodeship” [11]. The SWOT analysis for the key technologies of the region was performed, and scenarios and road maps of the technological development of the region until 2020 were compiled. The application of such a systematic approach enabled defining the technological portfolio of the Silesian Voivodeship within the foresight project.

The effect of the regional innovation strategy [9, 10] and the foresight project findings was also the development and implementation of the “Technology Development Program of the Silesian Voivodeship for Years 2010–2020” launched in 2009 [12]. The status of particular technological fields of the region was identified and the areas of the regional technological specializations of the Silesian Voivodeship, including medicine, power engineering, and information and communication technology (ICT), were indicated based on these and previous studies [9, 10].

The primary condition of the regional smart specializations development is the enhancement of the cooperation between science and business sectors with support and contribution of the regional authorities. The further development of traditional regional branches, through implementation of the best available technologies, and progress in new, prospective technologies should result in gaining the competitive advantage in the global market.

The objectives of the activities undertaken within the regional smart specialization related to medicine include the provision of high quality medical care to the citizens of the region, which could be achieved mainly through the implementation of the advanced solutions of e-medicine, medical engineering, biotechnology, materials science, information technology, and electronic engineering.

The power industry is the key economic sector of the region and the entire country. Within this specialization, particular attention should be given to the activities in the field of safe and efficient production of coal and preparation of ultraclean coals for the power industry, especially new technological solutions for production of the final energy carriers, systems for coal conversion into energy carriers, including combustion technologies (supercritical and ultrasupercritical parameters), coal gasification and polygeneration, combined heat, electricity, and cold generation, coal-based technologies for combined steel and energy carriers production, hydrogen-based power industry and combustion of coal in small environment-friendly heating boilers, and mitigation of hazardous substance emissions (including carbon dioxide) from the processes of coal utilization. The development of energy efficient technologies and utilization of renewable energy resources were identified as prospective areas of innovative solutions implementation.

The key aspects in the field of the enhancement of the regional smart specializations related to the information and communication technologies include increasing the access to knowledge, by means of participation in global cooperation networks and creation of transaction and management system for smart markets, as well as implementation of technologically advanced solutions of material and electronic engineering.

The concordance matrices are tools for the identification of cross-sectional connections, niche areas in given sectors, and for the assessment of the specialization potential, including the effects of public intervention and their influence on the R&D, economic, and social domains.

In principle, concordance matrices are well known and have been widely studied in a number of recent papers and, most thoroughly, by global market organizations. The uniqueness of the approach presented in this study derives from the practical implementation of a detailed cross-sectoral analysis into the regional context by construction of science-economy-technology concordance matrices. Regional R&D&I actors, owing to concordance matrices-based analysis of R&D and business value chains, are able to redefine their research and market attitude by improvement of the existing technologies contributing to the domains of regional technological specializations of the Silesian Voivodeship. Understanding and valuing science-economy-technology interconnections improve adaptation abilities by integration of regional actors and clustering their potential. A synergy effect of those activities was embedded in regional strategic documents such as the Regional Innovative Strategy Implementation Model [13] and creates a solid framework for complementary and targeted public intervention supported by EU structural funds. The implementation of the evidence-based policy principles by circular analysis of transition processes expands the regional development capacities and increases the competitiveness of the Silesian Voivodeship.

The main objective of the study presented in the paper was the development and analysis of the science-economy-innovative technologies concordance matrices for the regional smart specializations of the Silesian Voivodeship.

## 2. Methods

### 2.1. Concordance Matrix: The Basis

The science-industry concordance matrices are constructed based on the methodology developed by the Organization for Economic Cooperation and Development [14]. The influence of the scientific activities on defining the scientific and technological policy of the region was taken into account in the construction of the concordance matrices. The basis of the analysis is the determination of the connections between R&D expenditures (Government Budget Appropriations or Outlays for Research and Development, GBAORD) for 14 socioeconomic objectives, according to NABS classification (Nomenclature for the Analysis and Comparison of Scientific Programmes and Budgets), economic activity data (EU Labour Force Survey, EU-LFS), and the statistical classification of the economic activity in the European Community, Rev. 2 (Nomenclature Statistique des Activités Économiques dans la Communauté Européenne; NACE Rev. 2). The connections between NABS activities (see Table 1) and NACE codes (see Table 2) are sought in the procedure of concordance matrix development.

### 2.2. Concordance Matrix: Method of Construction

The procedure for science-economy-technology concordance matrix construction proposed by the authors comprises six stages:

1. Recognition of the research and economic components of the potential regional specialization based on statistical data concerning economy, R&D, and patents.
2. Linking the economic, research, and technological components of the regional specialization with the technological innovation area by means of the international patent classifications.
3. Recognition of the potential share of key enabling technologies in the economic and technological dimensions of innovation.
4. Recognition of the potential contribution of high technologies (of high R&D intensity), except for key enabling technologies, to the economic and technological dimensions of innovation.
5. Recognition of the potential share of services of high R&D intensity in the economic dimension of innovation.
6. Final composition of the matrix based on the in-depth expert analyses with the application of the Delphi method.

The definition of the basic elements of the regional specialization, that is, the economic and research components, is of key importance in the approach presented. This stage (*first stage*) involves identification and description of the specialization based on the expert knowledge in the field concerned. The defined potential of specialization areas needs to be complemented with indicators providing information on the influence of the innovation on the economic development. This is accomplished in the* second stage* of the concordance matrix construction procedure consisting in the introduction of the IPC indicators (international patent classification). The World Intellectual Property Organization (WIPO) proposed the classification practice for the international/transregional technology comparison based on these indicators [15] and with the application of the NACE codes. The most updated classification, ISI-OST-INPI, involves technological areas of electrical engineering, instruments, chemistry, process engineering (material engineering), and mechanical engineering.

In each of the abovementioned areas, some subareas may be further distinguished. The classification facilitates the analysis of the technological development structures, regional, national, and international comparisons, and the determination of smart specialization profiles.

The third, fourth, and fifth stages cover the assessment of the regional potential in categories of knowledge economy with the application of the data acquired in the first stage and logical construction of the second stage.

The* third stage* is subdivided to cover two aspects.

*Aspect 1.* The first aspect is the recognition of patenting activity of the regional economy sectors in KET areas.

Data on patent applications in KETs category submitted during the few last years (codes, number of applications) by the entities located in the given region as well as data on patents of KET category awarded in the few recent years to the entities located in the given region is ordered according to NACE codes of the entities submitting patent applications or being awarded the patents. In this way, the information on economy sectors and types of entities generating technological innovations in KET areas (utilization of data obtained in the first and second stages) is acquired.

KET codes mentioned in the previous section may be found in concordance matrices IPC-ISIC (four-digit codes) and ISIC-IPC (four-digit codes). In this way, the information on economy sectors for potential implementations of KET is obtained. Next, the regional potential of these economy sectors is assessed (according to the distinct criteria).

It may happen that the entities of the given region have not demonstrated any activity in the KET areas or that the activity has been minor or ineffective. This does not, however, overrule aspect 2 of the analysis.

*Aspect 2.* The second aspect is the recognition of the potential demand of the regional economy sectors in terms of the innovations in KET areas.

The economy sectors considered to be the leading ones in the region (based on other criteria, e.g., values of location indicators and dynamics of the added value increase) are assessed in terms of the potential demand for KET technologies implementation, based on the concordance matrices IPC-ISIC and ISI-IPC (four-digit level).

The utilization potential for KET in smart specializations may be considered significant when the analysis results may be positively interpreted at least in one of the two aspects.

The* fourth stage* is performed, similar to the third stage, in two aspects.

*Aspect 1.* The first aspect is the recognition of the patenting activity of the regional economy sectors in high-tech area.

Data on patent applications in high-tech area submitted during the few last years (codes, number of applications) by the entities located in the given region as well as data on patents in high-tech category awarded in the few recent years to the entities located in the given region is ordered according to NACE codes of the entities submitting patent applications or being awarded the patents. In this way, the information on economy sectors and types of entities generating technological innovations in high-tech area (utilization of data obtained in the first and second stages) is acquired.

High-tech codes mentioned in the previous section may be found in concordance matrices IPC-ISIC (four-digit codes) and ISIC-IPC (four-digit codes). In this way, the information on economy sectors for potential high-tech implementations is obtained. Next, the regional potential of these economy sectors is assessed (according to the distinct criteria).

It is possible that the entities of the given region have not demonstrated any activity in the high-tech area or that the activity has been minor or ineffective. This does not, however, overrule aspect 2 of the analysis.

*Aspect 2.* The second aspect is the recognition of the affiliation of the leading regional economy sectors to high-tech industries.

The economy sectors considered to be the leading ones in the region (based on other criteria, e.g., values of location indicators and dynamics of the added value increase) are assessed in terms of the affiliation to high-tech industries. The high-tech potential may be considered significant when the analysis results may be interpreted positively at least in one of the two aspects.

The most favorable situation in terms of the regional policy (in case of both the third and the fourth stages) is when the economy sectors active in patenting and sectors of potential patent implementations are at the same time the leading sectors of the regional economy, indicated by the results of the first and second stages.

The analysis performed within the* fifth stage* is based on concordance matrices matching the economy sectors to KIA and KIABI (Knowledge-Intensive Activities: Business Industries) categories according to Eurostat high-tech industry and knowledge-intensive services [17].

The potential is assessed based on the analysis in two aspects.

*Aspect 1.* The first aspect is the recognition of the total share of knowledge-intensive services (including KIA and KIABI) in the regional economy potential (utilization of data acquired in the first and second stages).

*Aspect 2.* The second aspect is the recognition of the affiliation of leading (or declared as smart specializations) regional economy sectors of high R&D intensity services.

The potential share of knowledge-intensive services in the regional development may be considered significant when the analysis results may be interpreted as positive at least in one of the two aspects.

The* final stage* of concordance matrix construction is the expert study with the application of the Delphi survey [18–21], providing a competent, group opinion of the selected specialists of the considerable expertise in the thematic field concerned. A standard Delphi survey may be described as the study managed by the monitoring group, involving several stages of a questionnaire addressed to the selected anonymous experts, and aimed at the subjective and intuitive consensus. The distinctive feature of the Delphi method is the consideration of the results of the previous stage of the survey in the following assessment. The Delphi survey is divided into several research theses, with assigned questions and a set of predefined answers. The implementation of several stages of the survey allows for the modifications of the questionnaire, depending on the results of the previous stage, and the anonymous feedback to the experts on the outputs of the previous step of the poll. The respondents are given the opportunity to compare their own opinions with the views of other experts and to modify their assessments in the following stage of the survey to build a certain consensus.

A Delphi survey was conducted within two rounds as a part of a broader national study [22] involving four regions of Poland varying significantly in terms of the level of economic development, that is, the Silesian Voivodeship, the Masovian Voivodeship, the Łódź Voivodeship, and the Podlaskie Voivodeship. For this purpose, a profiled questionnaire (Excel sheet) was developed and tested throughout piloting phase and finally distributed among 203 (each round) selected experts representing R&D, industry, administration, and business supported organizations of the Silesian Voivodeship. Delphi questionnaires were distributed via e-mails and additionally the questionnaire in a form of downloadable Excel file was available on the project website. Detailed instructions and a helpline operated by project team were established to support respondents. The first round of the survey was conducted in September and the second round in October 2013. The first round had a response rate of 47%, while the second round had a response rate of 40%.

The objective of the Delphi survey applied in the concordance matrix construction presented in the paper was the identification and assessment of the strongest connections between science, economy, and key technologies of the region. Experts were asked to rank those connections by using a 5-point Likert scale ranging from 1 (not at all important) to 5 (absolutely essential). The consensus regarding the strength of the abovementioned connections was attained when more than 50% of expert responses agreed. The role of experts is especially important in the adaptation of base matrices. The results of the Delphi survey may either confirm or reject the connections presented in the initial matrix. They may also complement it with additional components, resulting from the specific characteristics of the regional smart specialization. The regional smart specialization concordance matrix for the Silesian Voivodeship was supplemented with the estimation of the strength of mutual relations, based on the selected statistical indicators adapted to the regional level.

## 3. Results and Discussion

The developed methodology of concordance matrix construction was implemented in the characterization of the regional smart specializations of the Silesian Voivodeship in the field of medicine, power industry, and ICT.

### 3.1. Construction of the Regional Smart Specialization Concordance Matrix: Medicine

The first step of the development of the smart specialization concordance matrix for the Silesian Voivodeship in the field of medicine was its adaptation to the socioeconomic objectives according to the NABS classification (see Table 1). The socioeconomic objective, 7: health, was selected, involving R&D activities related to the human healthcare, promotion and restoring, including nutrition and food hygiene, preventive medicine, also in the field of medical and surgeon treatment, hospital and domestic care, social medicine, including pediatrics and geriatrics, prevention and control of contagious and noncontagious diseases, monitoring of health conditions, occupational healthcare, public healthcare regulations, and healthcare services for high risk groups and distressed people [23].

In the next step, the identified socioeconomic objective was related to the NACE codes of sections C (manufacturing) and Q (human health and social work activities) (see Table 2).

The following step consisted in finding the connections between the identified regional smart specialization related economy branches and key enabling technologies based on the EU report [24]. In the report, the contribution of patents to particular industrial sectors, by KETs, that is, industrial biotechnology, micro- and nanoelectronics, advanced materials, photonics, nanotechnology, and advanced manufacturing systems, was presented. Based on this document, the technologies of the highest patent contribution (minimum 25%) to the medicine related economy sectors were identified [25] (see Table 3). These included industrial biotechnology, advanced materials, and advanced manufacturing systems identified as the result of the first stage, being a part of a concordance matrix construction method.

The key element of concordance matrix construction is linking the identified patents with the socioeconomic objectives (NACE). This was done based on the concordance table presented by Atmaca [26] and translating the NABS classification and NACE codes into the IPC indicators regarding manufacturing. This table is essential in the construction of concordance matrix since there are no listings which would take into account all economy sectors specified in the NACE codes. The list of the IPC numbers related to the economy sectors of the strongest linking to medicine is presented in Table 4 [26].

Based on the above procedure, the assessment of the patents of the strongest effect on the economy sectors related to medicine was made, as well as the evaluation of the actual status of these interrelations in the Silesian Voivodeship, based on the number of the patents awarded and patent applications of respective codes as a part of the second-stage analysis.

The recognition of the potential contribution of a given technology of high R&D intensity, other than KETs, to the economic and technological dimensions of innovation was performed based on the WIPO concordance tables, taking into account the technological areas and the respective patent codes.

The connectivity analysis was performed based on the selection of the technological areas of the strongest connection to medicine and respective patents (see Table 5). The selected patent codes correspond to the medical and biotechnological technologies and pharmaceutical preparations indicated in Table 4 and enable identifying the patents of the strongest effect on the development of the medicine related technologies. They may be therefore considered as a tool for the assessment of the actual development status of these technologies in the region, by means of matching the numerical values to the respective patent codes.

The subsequent step of the medicine related concordance matrix development was the recognition of the potential of high R&D intensity services in the economic dimension of innovation. The results of the first stage of the Delphi survey enabled determining the connections between the medicine-linked selected areas of science and economy. The science domains, the progress in which should contribute to the more effective economic development related to the regional smart specialization of the medicine, are given in Table 6. The scientific domains were identified according to the OECD classification.

The scientific areas related directly to medicine of the strongest influence on the economic fields were identified based on the results of the first stage of the Delphi survey. The significant interaction strength was assumed to be at least 4, in 5-degree scale of interaction. The identified relations presented in Table 7 indicate the science domains which should be developed in the region to stimulate a considerable economic growth in the priority areas defined by the regional smart specialization.

The identification of the economic activities most closely related to the medicine and of high R&D intensity was also made. The economic activity domains were characterized within four categories: high, medium-high, medium-low, and low technologies. They were also assessed to be highly knowledge-intensive activities [16]. The connectivity analysis of the economic activity sectors and the medicine as a regional smart specialization is presented in Table 8.

**Figure 1:**
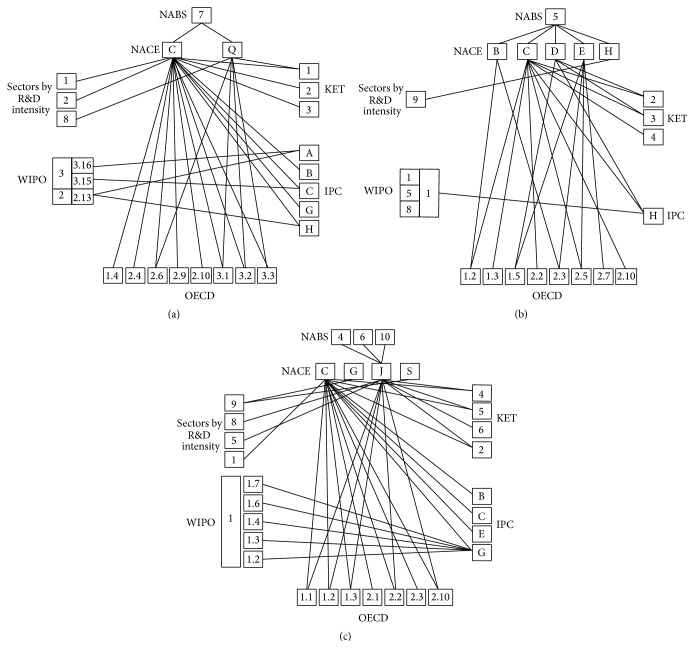
Concordance matrix for regional smart specialization in the field of (a) medicine, (b) power engineering, and (c) ICT.

The results of the above analysis of relations between science, economy, technology, and patent sectors in the field of the regional smart specialization are presented in the form of a concordance matrix in Figure 1(a).

### 3.2. Construction of the Regional Smart Specialization Concordance Matrix: Power Engineering

The regional smart specialization, power engineering, was assigned to the socioeconomic objective, 5: energy, according to the NABS classification given in Table 1, in the first step of the relevant concordance matrix construction. This objective includes R&D activities related to the manufacturing, storage, transportation, distribution, and rational utilization of all energy forms, processes focused on more efficient generation and distribution of energy, energy conservation and efficiency, carbon capture and storage, renewable energy resources, nuclear fission and fusion, fuel cells, and the remaining energy technologies [23]. The objective energy was related to the NACE codes (see Table 2) of section B (mining and quarrying), C (manufacturing), D (electricity, gas, steam, and air conditioning supply), E (water supply; sewerage, waste management, and remediation activities), and H (transportation and storage). The identified economy sectors related to the regional smart specialization considered were next analyzed against the connections with KETs. The technologies of the highest (at least 15%) contribution of patents to the energy sector were identified. The linking between the NABS socioeconomic objective, energy, NACE codes, and KETs for the regional smart specialization of power engineering is presented in Table 3. These dependencies were confirmed in the first stage of the Delphi survey. The strongest relations were identified for the science domains (according to the OECD classification) related directly to power engineering (electrotechnology, electronics, and information engineering): micro- and nanoelectronics, nanotechnology, photonics, and advanced power generation systems. In the following step of the study, the linking between the identified patents and socioeconomic objectives (NACE) for the regional smart specialization of power engineering was identified. The patents of two IPC classification groups were selected: H01 (basic electric elements) and H02 (generation, conversion, or distribution of electric power). The detailed list of IPC indicators for patents related to the economic sectors of power engineering is given in Table 4.

The recognition of the potential contribution of technologies of high R&D intensity, other than KETs, to the economic and technological dimensions of innovation was necessary for the construction of the complete concordance matrix. The patents relevant to the technological sectors most closely related to power engineering were identified based on the concordance tables developed with the application of the WIPO classification procedure. The assigned patent codes correspond to the IPC patent list (see Table 4). The list of patents specified for the power engineering sector was verified against the specialization specificity of the Silesian Voivodeship (see Table 5). The following patents of the strongest effect on the technology development, being the tool for the assessment of the actual power engineering technologies development in the Silesian Voivodeship, were identified within the technological sectors: machines, apparatuses, energy, semiconductors, and basic communication processes:

- Basic electric elements (H01).
- Generation, conversion, or distribution of electric power (H02).
- Basic electronic circuity (H03).
- Electric communication technique (H04).
- Electric techniques not otherwise provided for (H05).
- Semiconductor devices and electric solid state devices not otherwise provided for (H01L).

The first stage of the Delphi survey, concerning the influence of the science areas on the economic development of the region in the field of power engineering, enabled determining the respective types of economic activity according to the NACE classification and identifying the science domains of the strongest correlation with the given economic activities. Eight science domains, in which development would contribute to the more effective economic growth in the field of power engineering, were identified within the Delphi analysis (see Table 6). Five-degree interaction scale was applied in order to specify the economy sectors, highly influenced by the science domains directly related to power engineering, in the opinion of the experts of the first stage of the Delphi survey. The interaction strength of 4 was assumed to be significant. The identified dependencies of the economy sectors effect on the science domains directly related to power engineering are presented in Table 7. The identified interrelations indicated the science domains which should be advanced in order to facilitate the economic development in the field of power engineering. The service sector of high R&D intensity was identified in the process of concordance matrix construction for the power engineering sector in the region analyzed (see Table 8). The concordance matrix constructed based on the analysis of the interrelations between science, economy, technology, and patents in the field of regional smart specialization of power engineering is presented in Figure 1(b).

### 3.3. Construction of the Regional Smart Specialization Concordance Matrix: Information and Communications Technologies

The information and communication technologies are one of the regional smart specialization areas of the Silesian Voivodeship of the strategic importance in terms of the technological, economic, and social development in the region. The socioeconomic objectives of 4: transport, telecommunication, and other infrastructure, 6: industrial production and technology, and, to a lesser extent, 10: culture, recreation, religion, and mass media were identified as the ones most closely connected to ICT. These objectives comprise R&D activities concerning the infrastructure and territorial development, including construction of buildings, land use planning, and protection against the negative effects of urban and rural planning. Furthermore, the R&D activities related to the transportation and telecommunication systems, civil engineering and water supply, facilitation of industrial production and technology, and manufacturing are also concerned. The translation of the specialization area into socioeconomic objectives enabled linking the latter ones with the NACE codes applied in the statistical classification of the economic activity in the EU (see Table 2). The analysis of these connections indicated the interrelations between the selected NABS objectives and the NACE codes of section J (information and communication). Additionally, the groups of NACE codes linked with ICT in sections C, G, and S (manufacturing, wholesale and retail trade, repair of motor vehicles and motorcycles, and other service activities, resp.) were identified, for which the concordance tables determining the areas linked with the NABS socioeconomic objectives are not available. The relations between the KETs and economy areas related to the regional smart specialization in the field of ICT were determined based on the patent share in the industrial sectors, by key enabling technologies, that is, industrial biotechnology, micro- and nanoelectronics, advanced materials, photonics, nanotechnology, and advanced manufacturing technologies. The identified key enabling technologies of the highest patent contribution (at least 25%) to the economy sectors related to ICT were micro- and nanoelectronics, nanotechnology, photonics, and advanced manufacturing technologies (see Table 3) [25].

The interrelations between the NABS socioeconomic objectives, NACE codes, and KETs for the regional smart specialization in the area of ICT, specified in Table 3, were confirmed by the results of the first stage of the Delphi survey, where the strongest link between the science domains related to ICT according to the OECD classification (i.e., computers and information technology, electrotechnology, and electronics and information engineering) was reported for micro- and nanoelectronics, nanotechnology, photonics, and advanced manufacturing technologies. The results prove that the progress within these scientific fields is of the strongest effect on the implementation of the key enabling technologies.

The identified patents were next linked to the socioeconomic objectives. The concordance plot was developed based on the methodology adopted and with the application of the concordance tables, enabling the determination of patents of the strongest influence on the economy sectors related to ICT [26]. The actual status of these dependencies in the Silesian Voivodeship was also assessed based on the number of patents awarded and patent applications of the codes indicated for the ICT area. The list of the IPC patent numbers related to the economy sectors most closely linked to ICT is presented in Table 4.

The selection of the technologies of high R&D intensity, other than KETs, in the economic and technological dimensions of innovation was based on a WIPO concordance table, taking into account the technological areas and the patent codes assigned, respectively. The identified interrelations indicate the patents of the strongest effect on the advancements in technologies related to ICT (see Table 5) and may be applied as a tool for the assessment of the actual technology development status in the region by means of number values assigned to the selected patent codes.

The results of the first stage of the Delphi survey were applied in the search of the dependencies between economic sectors and science domains for the regional smart specialization of ICT. Similar to other smart specialization areas, the science domains of the strongest correlation with the given areas of economic activities were indicated by the experts (at least 4 points awarded in the 5-degree scale). The science domains, according to the OECD classification, in which development should contribute to the more effective economy development related to ICT, are presented in Table 6.

The economy sectors of the major influence on science domains were also identified (see Table 7) by linking the selected OECD-classified science areas with the NACE codes. This enabled recognizing the science fields which should be developed to facilitate the economic progress in the area of the specialization concerned.

The final stage of the methodology developed for the regional smart specialization concordance matrix construction was the identification of the economy sector, manufacturing, of the strongest link with ICT and high R&D intensity, taking into account the categories, high, medium-high, medium-low, and low technologies, and knowledge-intensive economic activity areas within the service sectors related to the ICT. The connectivity analysis of economy activity sectors and regional smart specialization in the area of ICT is presented in Table 8. The results of the presented methodology of the concordance matrix development for the regional smart specialization in the field of ICT are presented in Figure 1(c).

## 4. Summary and Conclusions

1. The comprehensive approach to the science-economy-technology concordance matrix construction developed within the study and presented on the examples of the regional smart specializations of the Silesian Voivodeship in the field of medicine, power engineering, and ICT proved to be an effective tool for the smart specialization potential assessment and for adequate addressing and evaluation of the support policies fostering the R&D&I sector.
2. A concordance matrix developed for the regional smart specializations facilitates accurate utilization of the expertise both at the stage of recognition of the significance/strength of the interrelations for the economic development and regional competitive advantage and at the stage of cyclical verification of the smart specialization strategy.
3. The procedure of concordance matrix construction presented in the paper is based on the European and world methodological output in the field concerned. It includes the recognition of the research and economic components of the regional specialization, linking the economic component of the regional specialization to the technological innovation area by means of the patent international classifications, recognition of the potential contributions of key enabling technologies and technologies of high R&D intensity, other than KETs, to the economic and technological dimensions of innovation, and the identification of the potential share of services of high R&D intensity in the economic dimension of innovation. It was deepened with the expert analyses performed within the Delphi survey.
4. The application of the procedure developed in the assessment of the interrelations between science, economy, and technology enables comparison and analyses of supraregional relations on the level of specialization related economy sectors and research areas.
5. Concordance matrices are useful tools for the development of the regional and national innovation policy based on smart specializations and implementation of the evidence-based policy principles to leverage potential of the regional innovation system by better addressed public intervention at multiple decision-making levels.
6. Concordance matrix construction approach provides a general understanding of the interrelation between regional value-chain components and potential of R&D&I sector being an immanent part of a cyclical assessment of the regional specialization strategies. Thus, concordance matrix construction procedure reinforced with a set of analytical tools has been implemented and provided as a web-based IT expert support system.

## Figures and Tables

**Table 1:** Socioeconomic activities, NABS classification.

Number	Code	Activity
1	NABS01	Exploration and exploitation of the earth
2	NABS02	Environment
3	NABS03	Exploration and exploitation of space
4	NABS04	Transport, telecommunication, and other infrastructure
5	NABS05	Energy
6	NABS06	Industrial production and technology
7	NABS07	Health
8	NABS08	Agriculture
9	NABS09	Education
10	NABS10	Culture, recreation, religion, and mass media
11	NABS11	Political and social systems, structures, and processes
12	NABS12	General advancement of knowledge: R&D financed from General University Funds (GUF)
	NABS13	General advancement of knowledge: R&D financed from sources other than GUF
13	NABS14	Defense

**Table 2:** Statistical classification of economic activities in the European Community, Rev. 2 (2008) [16].

NACE code number	Classification group
A01–A03	Agriculture, forestry, and fishing
B05–B09	Mining and quarrying
C10–C33	Manufacturing
D35	Electricity, gas, steam, and air conditioning supply
E36–E39	Water supply; sewerage, waste management, and remediation activities
F41–F43	Construction
G45–G47	Wholesale and retail trade; repair of motor vehicles and motorcycles
H49–H53	Transportation and storage
I55-I56	Accommodation and food service activities
J58–J63	Information and communication
K64–K66	Financial and insurance activities
L68	Real estate activities
M69–M75	Professional, scientific, and technical activities
N77–N82	Administrative and support service activities
O84	Public administration and defense; compulsory social security
P85	Education
Q86–Q88	Human health and social work activities
R90–R93	Arts, entertainment, and recreation
S94–S96	Other service activities
T97-T98	Activities of households as employers; undifferentiated goods- and services-producing activities of households for own use
U99	Activities of extraterritorial organizations and bodies

**Table 3:** NABS-NACE-key enabling technologies correspondence table for regional smart specializations in the field of medicine, power engineering, and ICT.

Regional smart specialization	NABS	NACE		KET
Medicine	7: health	C	21: manufacture of basic pharmaceutical products and pharmaceutical preparations	1: industrial biotechnology 2: advanced manufacturing technologies
			21.1: manufacture of basic pharmaceutical products	
			21.2: manufacture of pharmaceutical preparations	
			32.5: manufacture of medical and dental instruments and supplies	3: advanced materials
		Q	86: human health activities 86.1: hospital activities 86.2: medical and dental practice activities 86.9: other human health activities	1: industrial biotechnology
			87: residential care activities 87.1: residential nursing care activities 87.2: residential care activities for mental retardation, mental health, and substance abuse 87.3: residential care activities for the elderly and the disabled 87.9: other residential care activities	—

Power engineering	5: energy	B	5: mining of coal and lignite	—
			6: extraction of crude petroleum and natural gas	
		C	27: manufacture of electrical equipment	3: advanced materials 4: micro- and nanoelectronics 5: photonics
			27.1: manufacture of electric motors, generators, transformers, and electricity distribution and control apparatus	
		D	35: electricity, gas, steam, and air conditioning supply	3: advanced materials 4: micro- and nanoelectronics
			35.1: electric power generation, transmission, and distribution	
			35.2: manufacture of gas; distribution of gaseous fuels through mains	
		E	38.2: waste treatment and disposal	—
		H	49: land transport and transport via pipelines	—
			49.5: transport via pipeline	

ICT	10: culture, recreation, religion, and mass media	J	58: publishing activities 58.2: software publishing	—
	4: transport, telecommunication, and other infrastructure		61: telecommunications 61.1: wired telecommunications activities 61.2: wireless telecommunications activities 61.3: satellite telecommunications activities 61.9: other telecommunications activities	5: photonics 6: nanotechnology
	6: industrial production and technology		62: computer programming, consultancy, and related activities 62.0.1: computer programming activities 62.0.2: computer consultancy activities 62.0.3: computer facilities management activities 62.0.9: other information technology and computer service activities	4: micro- and nanoelectronics 5: photonics 2: advanced manufacturing technologies
			63: information service activities 63.1: data processing, hosting, and related activities; web portals	—
		C	26: manufacture of computer, electronic, and optical products 26.1: manufacture of electronic components and boards 26.2: manufacture of computers and peripheral equipment 26.3: manufacture of communication equipment 26.4: manufacture of consumer electronics 26.8: manufacture of magnetic and optical media	4: micro- and nanoelectronics 5: photonics 2: advanced manufacturing technologies
	—	G	46: wholesale trade, except of motor vehicles and motorcycles 46.5: wholesale of information and communication equipment	—
	—	S	95: repair of computers and personal and household goods 95.1: repair of computers and communication	—

**Table 4:** List of IPC patent indicators related to the economy sectors of the strongest effect on the regional smart specialization in the fields of medicine, power engineering, and ICT.

Regional smart specialization	Patents	NACE
Medicine	A61K, A61P, C07D, C07H, C07J, C07K, C12N, C12P, and C12Q	21: manufacture of basic pharmaceutical products and pharmaceutical preparations 21.1: manufacture of basic pharmaceutical products 21.2: manufacture of pharmaceutical preparations
	A61B, A61C, A61D, A61F, A61G, A61H, A61J, A61L, A61M, A61N, A62B, B01L, B04B, B29C, B29D, C12M, G01T, G21G, G21K, and H05G	32.5: manufacture of medical and dental instruments and supplies
	—	86: human health activities 86.1: hospital activities 86.2: human health activities 86.9: other human health activities
	—	87: residential care activities 87.1: residential nursing care activities 87.2: residential care activities for mental retardation, mental health, and substance abuse 87.3: residential care activities for the elderly and the disabled 87.9: other residential care activities

Power engineering	H01, H02 (H01H, H01R, H01M, H01K, H01T, H02K, H02N, H02P, H02B, H02H, and H02M)	27: manufacture of electrical equipment 27.1: manufacture of electric motors, generators, transformers, and electricity distribution and control apparatus
		35: electricity, gas, steam, and air conditioning supply 35.1: electric power generation, transmission, and distribution 35.2: manufacture of gas; distribution of gaseous fuels through mains
	—	49.5: transport via pipelines

ICT	B24D, B28B, B28C, B32B, C03B, C03C, C04B, E04B, E04C, E04D, E04F, and G21B	26: manufacture of computer, electronic, and optical products 26.1: manufacture of electronic components and boards 26.2: manufacture of computers and peripheral equipment 26.3: manufacture of communication equipment 26.4: manufacture of consumer electronics 26.8: manufacture of magnetic and optical media

**Table 5:** List of patents related to the technology fields of the regional smart specialization in medicine, power engineering, and ICT, according to the WIPO classification [15] (the first stage).

Regional smart specialization	Patents	Technology field	
Medicine	A61B, A61C, A61D, A61F, A61G, A61H, A61J, A61L, A61M, A61N, and H05G	2: instruments	2.13: medical technology
	C07G, C07K, C12M, C12N, C12P, C12Q, C12R, and C12S	3: chemistry	3.15: biotechnology
	A61K		3.16: pharmaceuticals

Power engineering	H01 (H01B, H01C, H01F, H01G, H01H, H01J, H01K, H01M, H01R, and H01T) H02, H05B, H05F, and H99Z	1: machines, apparatus, and energy	
	H01L	8: semiconductors	
	H03	5: basic communication processes	

ICT	G06# not G06Q, G11C	1: electrical engineering	1.6: computer technology
	H04L		1.4: digital communication
	G08C, H01P, H01Q, H04B, H04H, H04J, H04K, H04M, H04N-001, H04N-007, H04N-011, and H04Q		1.3: telecommunications
	G09F, G09G, G11B, H04N-003, H04N-005, H04N-009, H04N-013, H04N-015, H04N-017, H04R, H04S, and H05K		1.2: audio-visual technology
	G06Q		1.7: IT methods for management

**Table 6:** Science domains contributing to the more effective economic development related to the regional smart specialization in medicine, power engineering, and ICT (the sixth stage).

Regional smart specialization	NACE classification	Science domains acc. to OECD
Medicine	Chemical, pharmaceutical, and cosmetic industries	1.4: chemical sciences
	Chemical, pharmaceutical, and cosmetic industries	2.4: chemical engineering
	Manufacture of medical equipment and instruments Medicine and medical activities	2.6: medical engineering
	Chemical, pharmaceutical, and cosmetic industries	2.9: biotechnology
	Chemical, pharmaceutical, and cosmetic industries	2.10: nanotechnology
	Chemical, pharmaceutical, and cosmetic industries Manufacture of medical equipment and instruments Medicine and medical activities	3.1: basic medicine
	Chemical, pharmaceutical, and cosmetic industries Manufacture of medical equipment and instruments Medicine and medical activities	3.2: clinical medicine
	Chemical, pharmaceutical, and cosmetic industries Manufacture of medical equipment and instruments Medicine and medical activities	3.3: health sciences

Power engineering	Mining and quarrying	1.5: earth and related environmental sciences 2.3: mechanical engineering
	Power generation, including renewable energy	1.5: earth and related environmental sciences 2.3: mechanical engineering 2.5: materials engineering 2.7: environmental engineering
	Electrical and electronic industry	1.6: computer and information science 1.3: physical sciences 2.2: electrical engineering, electronic engineering, and information engineering 2.5: materials engineering 2.10: nanotechnology
	Manufacture and supply of media (electrical power, gas, and water)	1.5: earth and related environmental sciences

ICT	Electrical and electronic industry, information and telecommunication	1.1: mathematics
	Electrical and electronic industry, information and telecommunication, and logistics	1.2: computer and information science
	Electrical and electronic industry, information and telecommunication	1.3: physical sciences
	Logistics	2.1: civil engineering
	Electrical and electronic industry, information and telecommunication, and logistics	2.2: electrical engineering, electronic engineering, and information engineering
	Electrical and electronic industry	2.3: mechanical engineering
	Electrical and electronic industry, information and telecommunication	2.10: nanotechnology

**Table 7:** Interrelations between economy and science sectors for regional smart specialization of medicine, power engineering, and ICT (the sixth stage).

Regional smart specialization	Science domains (OECD)	NACE classification
Medicine	Medical engineering	Manufacture of medical equipment and instruments Medicine and medical activities
	Basic medicine	Chemical, pharmaceutical, and cosmetic industry Manufacture of medical equipment and instruments Medicine and medical activities
	Clinical medicine	Chemical, pharmaceutical, and cosmetic industry Manufacture of medical equipment and instruments Medicine and medical activities
	Health sciences	Chemical, pharmaceutical, and cosmetic industry Medicine and medical activities

Power engineering	Physical sciences	Power generation, including renewable energy Electrical and electronic industry
	Earth and related environmental sciences	Mining and quarrying Power generation, including renewable energy Manufacture and supply of media (electrical power, gas, and water)
	Electrical engineering, electronic engineering, and information engineering	Power generation, including renewable energy Electrical and electronic industry Information and telecommunication
	Environmental engineering	Power generation, including renewable energy

ICT	Computer and information science	Power generation, including renewable energy, manufacture of machinery, manufacture of weapons and ammunition, electrical and electronic industry, information and telecommunication, optical industry, logistics, manufacture of medical equipment and instruments, manufacture and supply of media, medicine and medical activities, and BPO
	Electrical engineering, electronic engineering, and information engineering	Power generation, including renewable energy, manufacture of machinery, manufacture of weapons and ammunition, electrical and electronic industry, information and telecommunication, transport (rail transport, air transport, transport by road, and manufacture of transport equipment), logistics, manufacture of medical equipment and instruments, manufacture and supply of media, civil engineering, creative activities, radio and television production, financial, insurance and real estate activities, and BPO

**Table 8:** Interrelations between economy activity based on classification of manufacturing and services by R&D intensity for regional smart specializations of medicine, power engineering, and ICT (the sixth stage).

Regional smart specialization	Sector	Category	NACE codes
Medicine	Manufacturing	1: high technology	21: manufacture of basic pharmaceutical products and pharmaceutical preparations
	Manufacturing	2: medium-high technologies	32.5: manufacture of medical and dental instruments and supplies
	Knowledge-intensive services	8: other knowledge-intensive services	86–88: human healthcare, residential care, and social work activities

Power engineering	Services	9: less knowledge-intensive services	49: land transport and transport via pipelines

ICT	Manufacturing	1: high technology	26: manufacture of computer, electronic, and optical products
	Knowledge-intensive services	5: high technology services	61: telecommunications 62: computer programming, consultancy, and related activities 63: information service activities
		8: other knowledge-intensive services	58: publishing activities
	Less knowledge-intensive services	9: less knowledge-intensive services	46: wholesale trade, except of motor vehicles and motorcycles
			95: repair of computers and personal and household goods
